# Unforgeable Red: Design and Application of SO‐Annulated Perylene Diimides for Anti‐Counterfeiting

**DOI:** 10.1002/smll.74331

**Published:** 2026-07-01

**Authors:** Pablo Simón Marqués, Sandra Pérez‐Domínguez, Nicolas Bréfuel, Jose M. Porro, So Ueno, Nathalie Saffon‐Merceron, Hiroko Yamada, Laurent Malaquin, Claire Kammerer

**Affiliations:** ^1^ Université de Toulouse, CNRS CEMES Toulouse France; ^2^ BCMaterials Basque Center for Materials Applications and Nanostructures Leioa Spain; ^3^ LAAS‐CNRS Université de Toulouse CNRS Toulouse France; ^4^ IKERBASQUE Basque Foundation for Science Bilbao Spain; ^5^ Institute for Chemical Research Kyoto University Uji Kyoto Japan; ^6^ Université de Toulouse Institut de Chimie de Toulouse, ICT UAR 2599 Toulouse France

**Keywords:** annulated‐perylene diimides, anti‐counterfeiting, photoresponsive materials, sulfoxides

## Abstract

The modernization of counterfeiting activities represents a threat to society, making difficult to detect and eliminate fraudulent products. In this context, innovation in anti‐counterfeiting technologies is crucial to protect intellectual property and increase the level of security. Herein, we report the first sulfoxide‐annulated perylene diimides (PDI‐SO) as versatile photoactive materials for advanced anti‐counterfeiting. Chemoselective mono‐oxidation of S‐bridged perylene diimides affords a red‐emissive PDI‐SO dye that undergoes rapid, quantitative visible light‐induced deoxygenation to give the green‐emissive PDI‐S. The materials retain high fluorescence efficiency, thermal robustness, and excellent processability, allowing patterning from the centimeter to micrometer scale using low‐cost masks, high‐resolution photolithography, and maskless photopatterning, and enabling dual optical readouts. Applications on textiles and electronic substrates coupled with molecular verification via swab‐mass spectrometry demonstrate that SO‐bridged PDIs provide a powerful, multi‐level, and difficult‐to‐replicate platform for secure authentication technologies.

## Graphical abstract

Sulfoxide‐annulated derivatives were synthesized as photoactive chromophores and implemented in photopatterning processes to design anti‐counterfeiting signatures with a double level of authentication. Patterns ranging from centimeter to micrometer size were obtained with high definition, and thereafter these approaches were tested in potential market niches such as fabric materials and electronic devices.

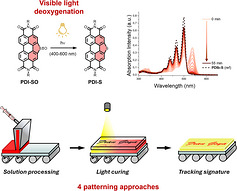

## 1 Introduction

The fight against forgery and counterfeit goods has drastically grown in recent years, driven both by the increasing sophistication of fraudulent technologies capable of producing high‐quality imitations and the challenges of tracing product origins in international trade. Accordingly, improving anti‐counterfeiting technologies is essential to protect the intellectual property of goods and to eliminate health risks associated with deceptive medicines or food [1, 2].

Within the anti‐counterfeiting strategies, luminescent patterns stand out over other approaches like watermarks or barcodes that can be readily duplicated. Luminescence anti‐counterfeiting affords a higher degree of security exploiting unique optical properties of the materials that hamper direct replication by simple printing methods while offering more reliable verifications [3, 4, 5, 6]. Quantum dots [7, 8, 9, 10, 11], perovskites [7, 11, 12], and lanthanide‐doped nanoparticles [13, 14, 15, 16] have been widely investigated as functional materials for luminescence anti‐counterfeiting. However, despite their outstanding emission properties, these systems may present some reproducibility issues and raise safety concerns connected to the presence of toxic compounds [17, 18, 19, 20, 21]. As an alternative, certain organic dyes [22, 23] provide similar photoluminescence with high reproducibility owing to the limited batch‐to‐batch variations in their synthesis [24, 25] and can be tailored to ensure biocompatibility [26]. Among them, photochromic compounds such as tetraphenylethylenes [27, 28, 29], spiropyrans [30, 31, 32] or azobenzenes [33, 34, 35], have attracted the attention of the community, since they enable the implementation of double level of verification, in contrast with single fluorescence materials, which are more susceptible to replication. Probably for this reason, perylene diimide (PDI) dyes have scarcely been used in anti‐counterfeiting signatures, despite their broad utility in photovoltaics [36, 37, 38], optoelectronic devices [39, 40, 41] or theranostics [42, 43, 44]. For instance, Abumelha et al. reported the preparation of anti‐counterfeiting signatures using fluorescent perylene monoimide‐doped silica [45], while Gou and co‐workers blended a bare PDI derivative with paraffin to obtain a composite presenting thermodynamic fluorescence with potential application in encryption and security patterns [46].

To expand the potential of the PDI derivatives in anti‐counterfeiting applications, we envisioned the development of strategies to evolve toward double or even multiple authentication levels. Motivated by the high stability, excellent processability, and photoluminescence of PDIs, we tackled the synthesis of novel photoactive PDIs for application in anti‐counterfeiting advanced materials. While the PDI block lacks intrinsic photochromism, rational functionalization of the scaffold provides an efficient route to confer photochemical activity [47, 48]. In this sense, the sulfinyl moiety triggered our attention owing to its tunable photoreactivity and potential applications in photopatterning and anti‐counterfeiting [49, 50, 51]. Thus, we hypothesized that sulfoxide bay‐bridged PDI derivatives could undergo photochemical deoxygenation, analogous to dibenzothiophene *S*‐oxides, which release atomic oxygen [O(^3^P)] under UV‐light to yield the corresponding dibenzothiophenes [52, 53, 54, 55]. We thus designed a synthetic route to PDI sulfoxides bearing different alkyl amide substituents. Their optical and photochemical properties were systematically investigated to assess the deoxygenation reaction in solution and solid state. The resulting materials were implemented in the preparation of anti‐counterfeiting patterns via multiple techniques. Notably, the compounds exhibited distinct fluorescence responses before and after irradiation, enhancing the verification levels, while also demonstrating compatibility with different dimensions and motifs. Eventually, we evaluated the applicability of this technology to relevant market niches, highlighting the potential of the SO‐annulated PDIs as versatile materials for advanced anti‐counterfeiting signatures.

## 2 Results and Discussion

**SCHEME 1:**
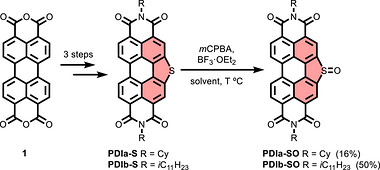
Synthetic route for the preparation of **PDIa‐SO** and **PDIb‐SO**. Conditions for the oxidation step: chlorobenzene, 70°C for **PDIa‐SO**; dichloromethane, ‐10°C for **PDIb‐SO**.

Novel sulfoxide‐bridged PDI derivatives were synthesized for the first time via controlled oxidation of the corresponding *S*‐annulated analogues. To mitigate strong π–π stacking interactions of the perylene blocks that typically lead to aggregation‐caused fluorescence quenching, bulky alkyl substituents, such as cyclohexyl and 6‐undecyl groups, were introduced on the imide moiety, and the corresponding sulfur‐bridged PDI compounds (PDI‐S) were obtained in three steps from perylene‐3,4,9,10‐tetracarboxylic dianhydride (**1**) according to previous reports in the literature [56, 57]. Successful oxidation of the thioether moiety to sulfoxide by means of *m*CPBA required the addition of BF_3_·OEt_2_ as a reaction additive, to prevent over‐oxidation and the formation of the more favoured sulfone as the major product (Scheme 1) [58, 59]. On the one hand, dicyclohexylimide **PDIa‐SO** was obtained after reaction optimization (see Supporting Information, section 2.1) in low yield (16%) due to the poor solubility of **PDIa‐S** precursor that needed to be heated at 70°C in chlorobenzene to start the reaction, thus compromising the kinetic control. In contrast, **PDIb‐S** carrying 6‐undecyl substituents displayed excellent solubility in chlorinated solvents and was smoothly oxidized at low temperature (‐10°C), which improved the reaction yield up to 50%.

**FIGURE 1:**
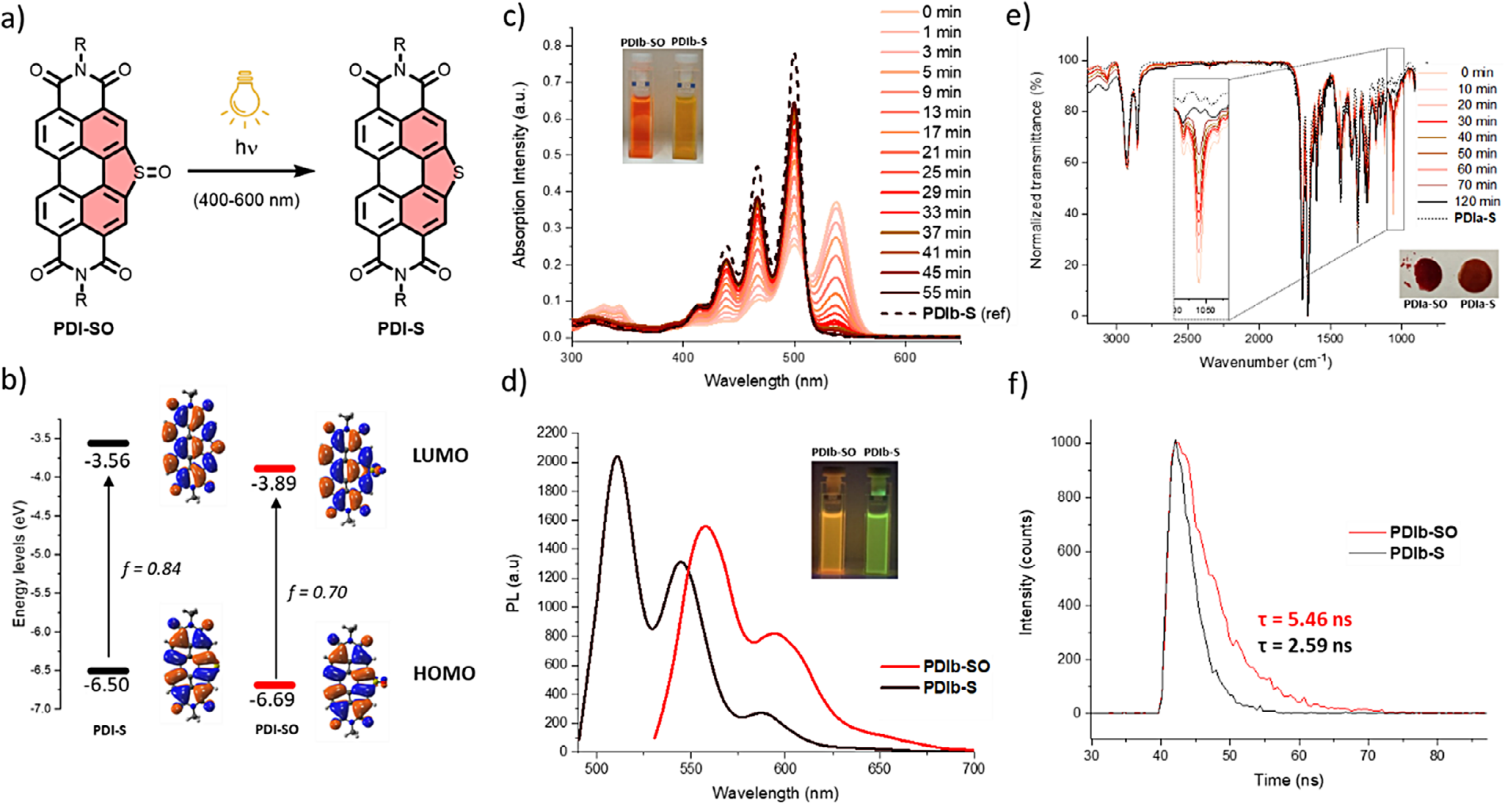
a) Photoinduced deoxygenation of **PDIa‐SO** and **PDIb‐SO** compounds to give as products the corresponding S‐annulated PDIs. b) Electron density and energy levels of the HOMO and LUMO calculated by DFT for the model systems **PDI‐S** (left) and **PDI‐SO** (right) bearing methyl substituents on the imides (R = Me). The oscillator strength *f* was calculated by TD‐DFT. c) Deoxygenation of **PDIb‐SO** in CH_2_Cl_2_ solutions (ca. 10^−5 ^M) upon visible light irradiation (400‐600 nm), monitored by UV–vis absorption spectroscopy. d) Photoluminescence spectroscopy in CH_2_Cl_2_ solutions (ca. 10^−6 ^M) of **PDIb‐SO** (red curve, λ_ex_ = 535 nm) and **PDIb‐S** (black curve, λ_ex_ = 490 nm). e) Deoxygenation of **PDIa‐SO** dispersed in KBr pellets (inset) upon visible light irradiation (400‐600 nm), monitored by IR‐spectroscopy and magnification of the sulfoxide fingerprint at ca. 1050 cm^−1^. f) Luminescence decay of **PDIb‐SO** and **PDIb‐S** in CH_2_Cl_2_ solutions (ca. 10^−6 ^M) measured at 560 and 515 nm, respectively.

The bright red **PDIb‐SO** derivative showed a structured absorption profile in CH_2_Cl_2_ solutions (10^−5^ M) between 450 and 550 nm, with λ_max_ at 538 nm, corresponding to a band identical to that of **PDIb‐S**, red‐shifted by ca. 39 nm (Figure S8). Density functional theory (DFT) and time‐dependent (TD)‐DFT calculations attributed this bathochromic shift to the reduction of the E_gap_ resulting from stabilization of the HOMO and the LUMO levels. These orbitals are also involved in the first excited state of both derivatives that is characterized by the typical π–π* transition of the PDI cores (Figure 1b; Figure S11). Additionally, **PDIb‐SO** presented red emission, with a structured band between ca. 525 and 700 nm and a Stokes shift of ca. 634 cm^−1^ (λ_max_ = 557 nm, λ_ex_ = 535 nm) (Figure 1d; Figure S9). In contrast, the deoxygenated analogue **PDIb‐S** displayed green fluorescence with the structured band ranging from 500 to 625 nm and the maximum located at 510 nm. Importantly, both **PDIb‐SO** and **PDIb‐S** showed promising fluorescence quantum yields (Φ_f_) and lifetime decays (τ), highlighting their potential application as luminescence materials. For instance, **PDIb‐SO** showed an enhanced Φ_f_ = 0.80 and τ = 5.46 ns compared to **PDIb‐S** (Φ_f_ = 0.66; τ = 2.59 ns) (Figure 1f).

**FIGURE 2:**
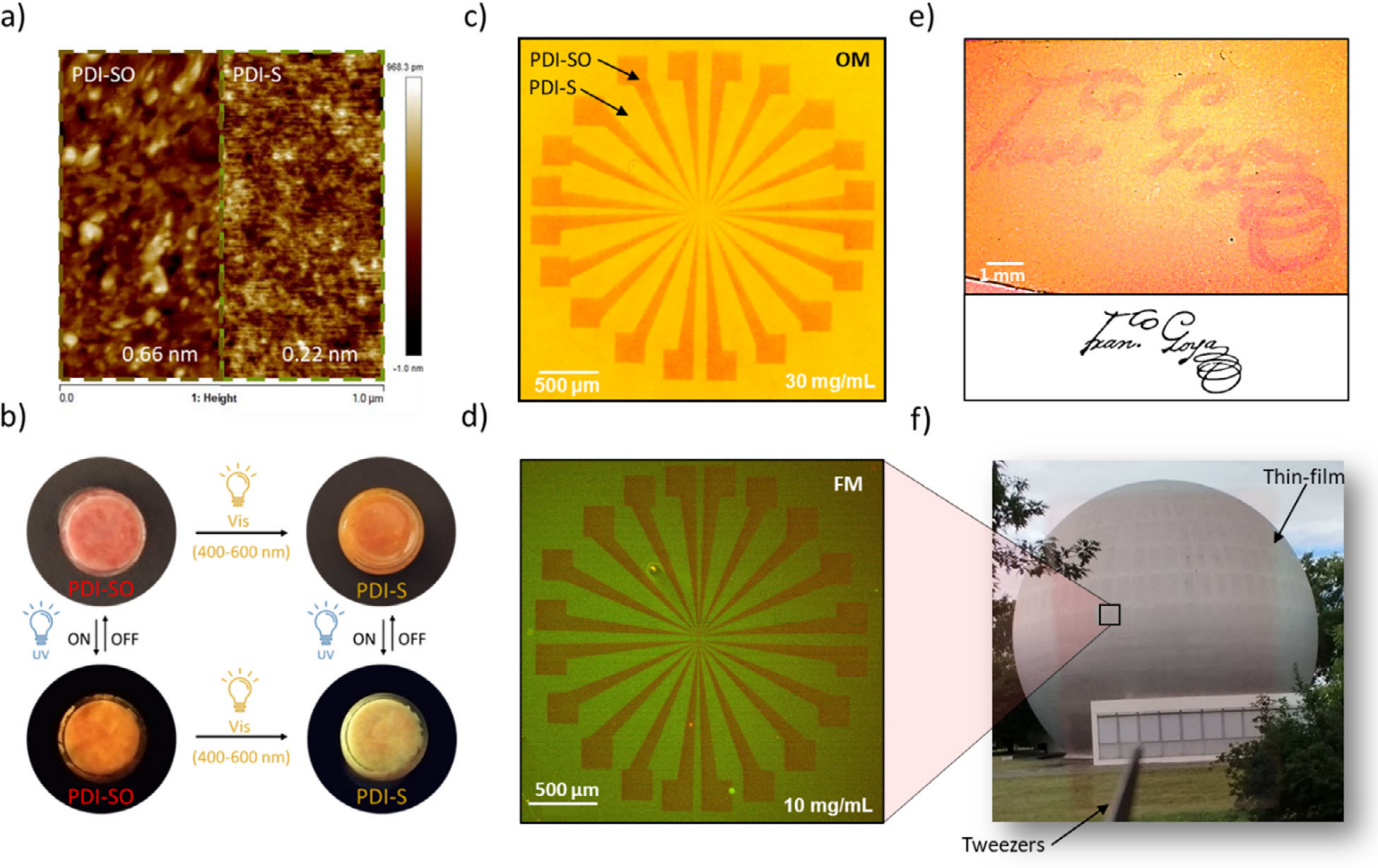
a) AFM micrographs of **PDIb‐SO** spin‐coated from CHCl_3_ (30 mg/mL, thickness = ca. 173 nm) onto glass before (left) and after (right) light irradiation. b) Double level luminescence anti‐counterfeit based on **PDIb‐SO**‐doped paraffin disc (Ø = 1.5 cm) observed under sun and UV light, before and after light curing using intense visible light (400‐600 nm). c) Optical micrograph (OM) of a **PDIb‐SO** anti‐counterfeiting pattern photoprinted through a chromium photomask onto a previously spin‐coated glass substrate. d) Fluorescence microscopy (FM) overlay of two images of a **PDIb‐SO** anti‐counterfeiting pattern using the 525 nm filter (green emission) and the 605 nm filter (red emission). e) Reproduction of signature of the painter Francisco de Goya, photoprinted onto a **PDIb‐SO** thin‐film using a handwritten mask. f) Day‐light photography of the CEMES‐CNRS [64] sphere through a transparent **PDIb‐SO** film (thickness = ca. 52 nm) deposited onto glass substrate (2 × 1.5 cm^2^) held by tweezers. In the thin‐film millimetric anti‐counterfeiting patterns are hidden and can be revealed by fluorescence microscopy (d).

Next, we explored the suitability of **PDIb‐SO** to undergo the photochemical deoxygenation of the sulfoxide moiety (Figure 1a), as reported for its smaller dibenzothiophene *S*‐oxide analogues. To our delight, irradiation of a CH_2_Cl_2_ solution with visible light (400‐600 nm) led to the quantitative conversion of **PDIb‐SO** into **PDIb‐S** in less than 1 h, contrasting with the low‐efficiency of deoxygenations previously reported for other dibenzothiophene derivatives [52, 54, 60]. The reaction was followed by UV–vis absorption spectroscopy (Figure 1c) showing the progressive disappearance of the **PDIb‐SO** maximum at 538 nm and the concomitant increase of the band at 499 nm, ascribed to the HOMO‐LUMO transition of the deoxygenated product **PDIb‐S**. The optical readout was translated into a clear color change of the solution from reddish to yellow, at the same time that the sample fluorescence drifted from orange to green (Figure 1d), perfect breeding ground for a double level anti‐counterfeiting technology. Transferring such in‐solution photochemistry to the solid state is always challenging, since restricted molecular mobility and tight packing in the solid state can hinder the conformational rearrangements and transient reactive encounters required for efficient photochemical transformations [61]. Thus, to evaluate the feasibility of this process in the solid state and confirm the potential of PDI‐SOs as photoactive organic materials, we first assessed the photochemical reaction of the insoluble **PDIa‐SO** embedded in KBr pellets, monitoring changes by infrared spectroscopy (IR) (Figure 1e). Since the elimination of oxygen only induces modifications in the sulfoxide group, the overall IR spectrum remained almost unaltered after curing the **PDIa‐SO** under visible light. Nevertheless, a thorough examination of the spectral fingerprints revealed a progressive attenuation of the characteristic peak in the S═O stretching region (1050 cm^−1^), until the signal overlapped the **PDIa‐S** reference spectrum, thus indicating the photoinduced oxygen release. To corroborate these observations, **PDIb‐SO** thin‐films were prepared by spin‐coating on different substrates to assess the light‐induced deoxygenation in a different environment and technique. The spin‐coated thin‐films showed an absorption comparable to the one in solution, red‐shifted by ca. 9 nm, consistent with the weakened π–π interactions between the perylene cores (Figure S10). Upon visible‐light irradiation, the sulfoxide deoxygenation succeeded smoothly, with a color change of the coated films from red to yellow that was monitored by UV–vis spectroscopy and confirmed by X‐ray photoelectron spectroscopy (XPS) as depicted in Figure S10 and Figure S28. After reaction completion, the **PDIb‐S** films presented photostability, showing no degradation even after 8 h of intense irradiation (Figure S10b). Optical microscopy techniques revealed no significant changes in the amorphous morphology after photodeoxygenation (Figure S24), while atomic force microscopy (AFM) micrographs depicted morphological changes such as the reduction of the nanodomain sizes, which resulted in a reduction of the film roughness from 0.66 nm to 0.22 nm (Figure 2a). Finally, we tested the thermal stability of the block by thermogravimetric analysis (TGA), which displayed no decomposition until ca. 290°C and the absence of thermal deoxygenation, thus supporting the envisioned application even under harsh environmental conditions (Figure S25).

To investigate the potential of the sulfoxide‐bridged PDIs in anti‐counterfeiting technologies, we aimed to assess their application in double luminescence responsive figures. As proof of concept, **PDIb‐SO**‐doped paraffin discs (10 mg/g) were fabricated (Figure 2b). The composite initially appeared pale red, with red‐orange emission when illuminated under a UV lamp. Following irradiation under intense visible light, the paraffin motifs changed color to yellow‐orange and the photoluminescence response to yellowish. In view of these promising results, we next explored the application of the new materials without the need of additives or any film‐forming agents, taking advantage of the well‐known processability of PDIs in the field of organic electronics. Thus, by combining its excellent solution processability and photoreactivity, **PDIb‐SO** was proposed for photoprinting anti‐counterfeiting patterns, offering superior reproducibility and resolution compared with fluorescence ink pens and printers.

To explore this idea, we first attempted to photoprint figures using handwritten masks, created by drawing the desired shape on a transparent surface with a dark marker (Figure S14). **PDIb‐SO** spin‐coated thin‐films were irradiated for ca. 30 min through the homemade mask, inducing photodeoxygenation and the corresponding color change of the illuminated regions, while the areas under the written shape retained their red color. As an example, a replica of Francisco de Goya's signature was successfully stamped onto a glass substrate coated with **PDIb‐SO**, as shown in Figure 2e. Using this sealing method, documents and goods can be marked with a personalized, irreproducible signature making use of inexpensive and accessible materials.

In order to prepare well‐defined patterns, the fabrication of new masks was carried out using a stereolithography 3D printer, which made possible to obtain computer designed motifs of photopolymerized resins. As shown in Figure S15, this method allowed us to photoprint the logo of CEMES‐CNRS [64] onto a 2 × 1.5 cm^2^ thin‐film, displaying sharply defined lines, corners and curves with a minimum feature size of 500 µm. However, the use of handwritten and 3D‐printed photomasks limited the resolution of the obtained patterns, and thus, to design smaller figures, alternative techniques had to be explored. In this sense, the use of chromium‐based masks on fused silica offered a higher resolution down to 50 µm of line width, suggesting virtually designed 2D patterns that can be computed in similar ways to the 3D printed designs. To test this approach, we used available masks bearing motifs previously used in electronics and the irradiated thin‐films were observed using a Dino magnification camera, revealing well‐defined figures of diverse nature, thanks to the color contrast between red **PDIb‐SO** and yellow deoxygenated **PDIb‐S** (Figure 2c; Figure S16). Additionally, the anti‐counterfeiting security of these patterns can be elevated to a double level by exploiting the photoluminescence properties of these compounds. Therefore, fluorescence microscopy (FM) revealed two different responses depending on the excitation wavelength and emission filter applied. When excited at ca. 560 nm and applying an emission filter limited to 580 to 625 nm, the patterns composed by non‐irradiated **PDIb‐SO** were the only responsive figures, attributed to the shifted emission of the SO‐annulated perylene. In contrast, exciting the thin‐film at ca. 490 nm, emission light was recovered from 500–550 nm making visible the negative of the previous patterns, where the deoxygenated **PDIb‐S** lies on. Overlaying the two fluorescence channels yielded a perfect match as depicted in Figure 2d and Figure S17. This concept was extended to transparent thin‐films obtained from diluted **PDIb‐SO** solutions, similarly to the preparation of transparent photovoltaics [62]. Though these films appeared limpid to the naked eye (Figure 2f), undetectable anti‐counterfeiting signatures were embedded on the substrate and were detected by fluorescence microscopy thanks to the enhanced response of the fluorescent materials. The patterns remained clearly visible even after a few months, though spectroscopic analysis revealed that slow deoxygenation starts after several days under ambient light conditions (Figure S10d).

**FIGURE 3:**
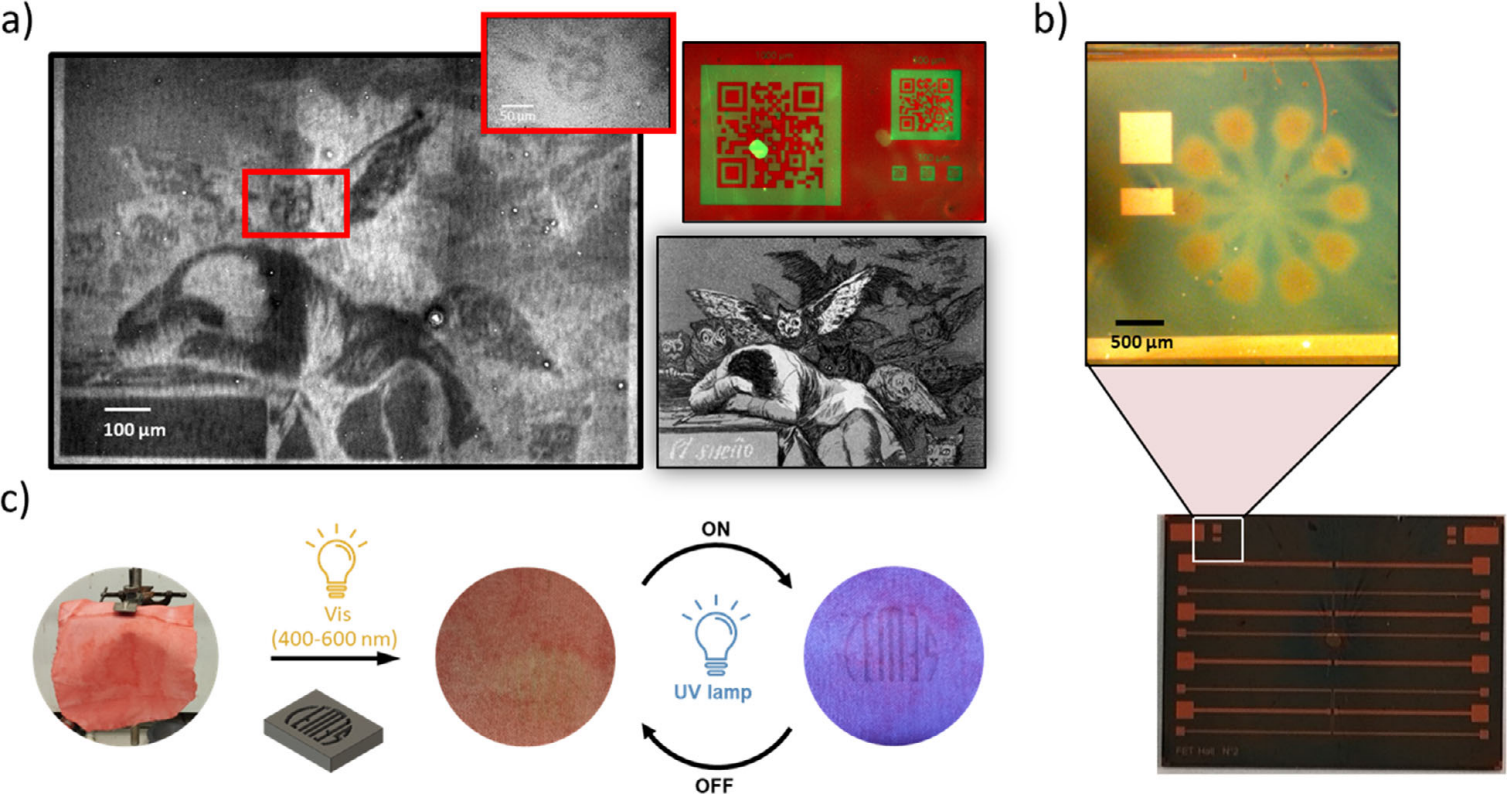
a) Motifs engraved onto a **PDIb‐SO** thin‐film by laser photopatterning. Top‐right, green and red channels micrograph of QR code micropatterns with 1000–100 µm square side visualized. The embedded link forwards to the CNRS homepage [63]. On the left, the photoprinted reproduction of Francisco de Goya's painting “The Sleep of Reason Produces Monsters”, with the magnification of the owl's head highlighting the high resolution. For comparison, Goya's original work is displayed in the right‐bottom corner. b) **PDIb‐SO**‐based OFET prototype bearing a star‐shaped motif stamped on it. c) Schematic process for dyeing and photoprinting anti‐counterfeiting signatures on fabric.

Eventually, to push the resolution limit, we explored a maskless photomicropatterning system equipped with a 405 nm light source capable of achieving a line width resolution of 3 µm at its highest performance, and thereby, opening doors to more complex designs on a reduced scale. This technology (PRIMO, Alveole) worked directly onto the **PDIb‐SO** thin‐film making use of a UV light source that is projected through a digital micromirror device (DMD) system, which directs the light onto the film in the shape of the designed pattern. UV light exposure can be modulated by increasing or decreasing the light dose applied. To illustrate this method, QR codes with square sides ranging from 1000 to 100 µm were imaged on the PDI thin‐film (Figure 3a), achieving well‐defined patterns visible under confocal microscopy, using 5x, 10x and 20x objectives, where the emissive contrast between **PDIb‐SO** and **PDIb‐S** became clear. Scanned with a standard mobile phone, the matrix successfully directs to the CNRS homepage (see Supporting Information Video) [63]. Thereafter, to assess the adequacy of the process to high‐resolution reproductions, a more complex image was attempted through the photopatterning process. We selected one of the most famous aquatints of the romantic painter Francisco de Goya, “The Sleep of Reason Produces Monsters”, which was photoprinted onto the photoactive material with dimensions of 1.8 × 1.3 mm^2^ (Figure 3a). Thanks to the original color of the aquatint in grey gradient areas, different light doses were exposed on the desired areas, leading to similar tones to those of Goya's original work. Most importantly, the figures and details portrayed in the thin‐film reached resolutions below 10 µm, as can be observed in the owl's head magnification.

Once proved the compatibility of the **PDIb‐SO** films with multiple photopatterning methods, we tried to strengthen the authentication scheme beyond double level by introducing a molecular readout. Thus, we evaluated the viability of conducting swab mass spectrometry (MS) tests to level up the degree of security. Since the signatures are composed of two pure compounds, **PDIb‐SO** and **PDIb‐S**, a profile containing the most abundant isotopes and adducts of the perylene derivatives should be observed in the obtained spectra. Similar to the airport security controls, a cotton swab was rubbed across a photoprinted film and subsequently analyzed by MS (MALDI‐TOF). As hypothesized, the resulting spectra revealed distinct signals of the perylene sulfoxide and its deoxygenated counterpart at 744.4 and 728.4 m/z, respectively (Figure S23). Incorporating this verification process renders falsification virtually impossible in the absence of a unique PDI‐SO ink, and although requiring additional analytical equipment, this strategy could be of great interest for goods of high‐value.

Eventually, we addressed the application of the anti‐counterfeiting technology in relevant market niches to demonstrate the potential transfer of our approach to real life. In this sense, the *fashion mode* and textile industry is the most affected sector in the European Union and one of the most affected globally. Among the different materials, cotton fabric triggered our attention, since the falsification of this bio‐based tissue can lead to health issues for customers with dermatological problems. Taking advantage of the compatibility of cotton with vat‐dying, a tissue square was dip‐dyed in a **PDIb‐SO** solution (Figure S19) followed by photoprinting through a 3D‐printed mask displaying the CEMES‐CNRS logo. Although no modifications were visible to the naked eye, the pattern was revealed when observed under the UV lamp, as depicted in Figure 3c. This approach offers the advantages of direct printing on fabric avoiding the use of additional materials, thus enabling the obtention of hidden signatures to verify authenticity of clothes.

Besides textiles, another area that triggered our attention is the field of electronics, which is experiencing significant growth and where counterfeit components can lead to severe system failures and security problems. **PDIb‐SO** proved highly compatible with organic electronics owing to the excellent processability and the possibility to print micro‐patterns. As an example, a **PDIb‐SO**‐based organic field effect transistor (OFET) prototype was engraved with an anti‐counterfeiting signature obtained using a chromium‐based mask (Figure 3b; Figure S20), demonstrating the potential of these materials in protecting high‐value electronic devices.

## 3 Conclusion

In conclusion, we report the first synthesis of SO‐annulated perylene diimides, along with the characterization of their photochemical behaviour and subsequent application of the resulting materials in anti‐counterfeiting. The PDI‐SOs were synthesized in 4 steps starting from commercially available perylenetetracarboxylic dianhydride, with the key reaction step being the chemoselective mono‐oxidation of the S‐bridged precursor mediated by *m*CPBA in the presence of BF_3_·OEt_2_. Evaluation of their optical and photochemical activity revealed significant modification of the absorption and emission properties upon light irradiation, which is of great value in encryption technologies.

The photoinduced deoxygenation of the PDI‐SOs was proven quantitative in solution and solid‐state, making use of visible light in the 400–600 nm range. Thereafter, the soluble **PDIb‐SO** was processed into thin‐films to design double‐level luminescence anti‐counterfeiting patterns by irradiation through a photomask. The fabrication process was compatible with different approaches, from low‐cost handwritten and 3D‐printed masks, to high‐resolution chromium masks or photomicropatterning technology. This versatility made it possible to cover a wide range of dimensions down to the micrometer scale and allowed the design of almost any kind of shape. Finally, the photoprinting processes were transferred to two different substrates, a **PDIb‐SO**‐dyed cotton fabric and an OFET prototype, to highlight the potential of these materials in relevant market niches. While these proof‐of‐concepts are highly promising, some challenges remain to be addressed prior to transfer in technological applications, such as adequate encapsulation materials, green processing techniques and patterning approaches compatible with automated and scaled processes.

[CCDC 2517519 contains the supplementary crystallographic data for this paper. These data can be obtained free of charge from The Cambridge Crystallographic Data Centre via https://www.ccdc.cam.ac.uk/data_request/cif.]

## Funding

This research has received funding from the French National Research Agency (ANR) through the France 2030 plan, and the Occitanie Region through the European Regional Development Fund (ERDF) (project LISaS, n°ANR‐22‐EXES‐0015). It has been supported by the French ANR with the project CROSS (n° ANR‐21‐CE09‐0002) and the project Printiss (n° ANR‐21‐CE19‐0041), and the Japan Society for the Promotion of Science (JSPS) KAKENHI Grant Numbers JP25K01751 and JP20H05833 (Transformative Research Areas “Dynamic Exciton”). The work was partly supported by the MultiFAB project funded by FEDER European Regional Funds and French Région Occitanie (16007407/MP0011594). P.S.M. and J.M.P. acknowledge Ramón y Cajal grants RYC2023‐045101‐I and RYC2023‐044841‐I funded by MICIU/AEI/10.13039/501100011033 and by FSE+. We also thank the CNRS for its support through the International Research Project POEMES (2024‐2028) and the International Short‐term Exchange Program for Young Researchers by the ICR‐iJURC, Kyoto University.

## Conflicts of Interest

The authors declare no conflict of interest.

## Supporting information




**Supporting File**: smll74331‐sup‐0001‐SuppMat.docx.

**Supporting File**: smll74331‐sup‐0002‐VideoS1.mp4.

## Data Availability

The data that supports the findings of this study are available in the supplementary material of this article and from the corresponding author upon reasonable request.
