# Supplementary material for: Unforgeable Red: Design and Application of SO‐Annulated Perylene Diimides for Anti‐Counterfeiting
Source: Small. 2026 Jul 1;22(46):e74331. doi: 10.1002/smll.74331 (PMC13485031; doi:10.1002/smll.74331)
Supplement: Supplementary file 1 [file SMLL-22-e74331-s001.docx]

**Unforgeable Red: Design and Application of SO-annulated Perylene Diimides for Anti-counterfeiting**

Pablo Simón Marqués,*^,a,b^ Sandra Pérez-Domínguez,^c^ Nicolas Bréfuel,^a^ Jose M. Porro,^b,d^ So Ueno,^e^ Nathalie Saffon-Merceron,^f^ Hiroko Yamada,^e^ Laurent Malaquin,^c^ Claire Kammerer*^,a^

a) Université de Toulouse, CNRS, CEMES, Toulouse, France

b) BCMaterials, Basque Center for Materials, Applications and Nanostructures, UPV/EHU Science Park, 48940 Leioa, Spain

c) LAAS-CNRS, Université de Toulouse, CNRS, Toulouse 31400, France

d) IKERBASQUE, Basque Foundation for Science, Bilbao, 48013, Spain

e) Institute for Chemical Research, Kyoto University, Gokasho, Uji, Kyoto 611-0011, Japan

f) Université de Toulouse, Institut de Chimie de Toulouse, ICT UAR 2599, 118 route de Narbonne, 31062 Toulouse, France

Supporting Information

[1. Materials and Methods	2](#_Toc230104888)

[1.1 Chemicals	2](#_Toc230104889)

[1.2 Methods	2](#_Toc230104890)

[2. Synthetic methods	3](#_Toc230104891)

[2.1 Synthesis of **PDIa-SO**	3](#_Toc230104892)

[2.2 Synthesis of **PDIb-SO**	6](#_Toc230104893)

[3. Optical characterization	10](#_Toc230104894)

[4. Theoretical calculations	11](#_Toc230104895)

[5. Anti-counterfeiting photopatterns	16](#_Toc230104896)

[5.1 Handwritten photomask	16](#_Toc230104897)

[5.2 Stereolithography 3D printing (polymer-based photomask)	16](#_Toc230104898)

[5.3 Micrometric patterns	17](#_Toc230104899)

[5.4 Fabric patterning	19](#_Toc230104900)

[5.5 OFET prototype patterning	19](#_Toc230104901)

[6. Swab test	20](#_Toc230104902)

[7. Solid state characterization	21](#_Toc230104903)

[7.1 Optical and polarized optical microscopy	21](#_Toc230104904)

[7.2 Thermogravimetric analysis	22](#_Toc230104905)

[7.3 Single crystal X-ray diffraction	22](#_Toc230104906)

[7.4 X-ray photoelectronic spectroscopy (XPS)	24](#_Toc230104907)

[7.5 Atomic force microscopy (AFM) analysis	26](#_Toc230104908)

[8. References	27](#_Toc230104909)

# 1. Materials and Methods

## 1.1 Chemicals

All chemicals listed herein were used as received without further purification if not indicated: perylenetetracarboxylic dianhydride, *m*CPBA (≤77wt%), BF_3_.OEt_2_, Na_2_CO_3_, Na_2_SO_4_, NH_4_OAc, NaBH_3_CN, HNO_3_, imidazole, cyclohexylamine, octadecane and sulfur were purchased from Merck. 6-Undecanone was obtained from ACROS.

## 1.2 Methods

**Reactions and purifications**: Reactions under inert atmosphere were carried out using standard Schlenk techniques. Flash chromatography was performed using an ARMEN-Interchim Glider Flash Spot 2 system and silicagel columns of 4, 12, 24 and 40 g from RediSep and puriFlash. Flexible plates ALUGRAM® Xtra SIL G UV254 from MACHEREY-NAGEL were used for TLC, with visualization effected using a UV lamp (254, 366 nm).

**Analysis**: ^1^H and ^13^C-NMR spectra were recorded on Bruker Avance III HD 500 MHz (cryoprobe Prodigy 5mm BBO, 1H ATMA), Avance 500 MHz (cryoprobe 5mm 1H, 13C) and Avance NEO 300 MHz (probe 5mm BBFO ATM) spectrometers. Chemical shifts (δ) are reported in ppm. Coupling constants (*J*) are given in Hz and the following abbreviations have been used to describe the signals: singlet (s); doublet (d); triplet (t); multiplet (m). Assignments of ^1^H and ^13^C NMR spectra were made with the assistance of HSQC spectra, as well as DFT NMR calculations (see section 4).

High‐resolution mass spectra (HR-MS) were performed with a Waters GCT Premier spectrometer for desorption chemical ionization (DCI-CH4). MALDI HR-MS and low-resolution MS were performed with a Bruker Tims Tof Flex spectrometer at Metatoul-Axiom (INRAE, UMR1331 Toxalim, Toulouse, France), MetaboHUB-ANR-11-INBS-0010.

UV–Vis spectra were recorded on a Varian Cary 5000 spectrometer and fluorescence spectra on a Hitachi F-4500 fluorescence spectrophotometer.

Infrared spectra (IR) were recorded with a Perkin-Elmer Spectrum 100 FT-IR, through KBr pellets containing the synthetic compound in 3% wt. For the preparation, the exact amounts of KBr and **PDIa-SO** were grounded in a mortar and the fine powder transferred to a hydraulic press to obtain the translucent KBr pellets after compacting the solids.

Thermogravimetric analysis (TGA) was carried out using an ATG/DSC3+ (Mettler-Toledo).

Atomic force microscopy (AFM) images were obtained in the tapping mode using an AFM Bruker D3000 with the basic phase extender module and a Park System NX10.

**Thin-film preparation**: Organic thin-films of compound **PDIa-SO** were prepared by spin-coating (6000 rpm, 1 min, Oscilla E441) from chloroform solutions (10-30 mg / mL) onto glass and quartz substrates.

**Paraffin disc:** **PDIb-SO** was dispersed in hot octadecane (10 mg/g) in a 3 mL vial and sonicated in an ultrasound bath until obtaining a homogeneous slurry. The mixture was cooled down till 5 ºC in the fridge and then taken to room temperature to obtain the solid paraffin disc with the vial bottom shape.

**Photoirradiation**: All photochemical experiments (unless otherwise stated) were carried with a HAMAMATSU UV Spot Light Source Lamp (L9588-06A) with a light guide and filter allowing 400-600 nm wavelengths. Light power and distance were adjusted to obtain an irradiation of 1200 mW/cm^2^. Kinetic experiments were conducted in a homemade photoreactor equipped with a visible light of 0.04 W/cm^2^.

**Light induced micropatterning (Primo):** QR codes and Goya’s painting patterns were done using a PRIMO equipment (Alvéole) with a 405 nm laser. PRIMO was installed in an Olympus IX71 microscope with a 4x objective (4X UPLANFL N) used for the generation of the patterns. Files were saved as .tiff files for the proper compatibility with LEONARDO software. Patterns were designed in black and white, allowing light to expose only white motifs.

**Microscopy:** Dino-Lite USB Digital microscope was used to capture magnified images of drawn patterns. Fluorescence observations were conducted using a ZEISS observer 7 microscope, employing 10x air objective using GFP filter set (excitation 470 ± 20 nm, emission 525 ± 25nm) and mCherry filter set (excitation 550 ± 12 nm, emission 605 ± 35 nm) and DMi8 Leica inverted microscope, employing 5x and 10x objectives using GFP filter set (excitation 470 ± 20 nm, emission 525 ± 25nm) and mCherry filter set (excitation 550 ± 10 nm, emission 600 ± 20 nm).

# 2. Synthetic methods

*Scheme S1. Synthetic route for the preparation of* ***PDIa-SO*** *and* ***PDIb-SO*** *target compounds.*

The synthesis of the annulated derivatives **PDIa-S** and **PDIb-S** was performed following procedures reported in the literature.^[1,2]^ 6-Aminoundecane was prepared from 6-undecanone as previously reported.^[3]^

## 2.1 Synthesis of PDIa-SO

A suspension of **PDIa-S** (30 mg, 51.3 µmol, 1 equiv.) in chlorobenzene (25 mL) was cooled to 0 °C and BF_3_·Et_2_O (52 μL, 59 mg, 0.41 mmol, 8 equiv.) was added. To the resulting mixture was added a solution of *m*CPBA (12 mg, 52.5 µmol, 1.05 equiv.) in chlorobenzene in several portions. The reaction was stirred at 70 ºC during 4 h, cooled to room temperature and then quenched by careful addition of water (5mL) and Na_2_CO_3_ (5 mL, 1.0 M). The organic phase was collected, dried over Na_2_SO_4_ and the solvent removed under rotatory vacuum. The crude was purified by flash chromatography (eluent: CH_2_Cl_2_/AcOEt, 99:1 to 9:1) to give **PDIa-SO** as a red solid (5 mg, 8.32 µmol, 16% yield). ^1^H NMR (300 MHz, CDCl_3_, 25 °C): δ (ppm) 9.08 (s, 2H, H_1_), 8.75 (d, *J* = 7.9 Hz, 2H, H_2_), 8.60 (d, *J* = 7.9 Hz, 2H, H_3_), 5.10 – 4.99 (m, 2H, H_4_), 2.62 – 2.50 (m, 4H, H_5_), 1.98 – 1.87 (m, 4H, H_6_), 1.81 – 1.71 (m, 6H, H_5_’/H_7_), 1.33 – 1.21 (m, 6H, H_6_/H_7_’). HRMS (MALDI-TOF-): *m/z* calcd for C_36_H_28_N_2_O_5_S 600.1724, found: 600.1736 [M^-^]. ^13^C NMR could not be recorded due to the insolubility of **PDIa-SO** in common organic solvents.

The above-mentioned conditions result from an optimization process that was carried out to overcome the insolubility of **PDIa-S**. Initially, the reaction involved low temperatures (see reaction conditions for the oxidation of **PDIb-S**), which resulted in the recovery of the starting material. The temperature was optimized up to 70 ºC, where the highest yield was obtained under these reaction conditions. Other deviations from the optimized conditions resulted lower and null yields as depicted in Table S1.

*Table S1. Different conditions used for the oxidation of* ***PDIa-S*** *to* ***PDIa-SO*** *with mCPBA.*

| **Entry** | **Variation from standard conditions** | **Yield** |
| --- | --- | --- |
| 1 | None | 16% |
| 2 | Room temperature instead of 70 ºC | - |
| 3 | Reflux of CH_2_Cl_2_ instead of PhCl | - |
| 4 | Tris(pentafluorophenyl)borane instead of BF_3_.OEt_2_ | - |

**H_6_**

**H_6_’**

**H_7_**

**H_5_’**

**H_5_**

**H_7_’**

**H_4_**

**H_1_**

**H_3_**

**H_2_**

*Figure S1. ^1^H-NMR spectrum of* ***PDIa-SO****, in CDCl_3_ (300 MHz, 25 °C).*

*Figure S2. High Resolution Mass Spectrometry spectrum of* ***PDIa-SO*** *(MALDI-TOF-).*

## 2.2 Synthesis of PDIb-SO

To a solution of **PDIb-S** (105 mg, 0.14 mmol, 1 equiv.) in CH_2_Cl_2_ (10 mL) at -10 ºC, BF_3_·Et_2_O (0.15 mL, 164 mg, 1.15 mmol, 8 equiv.) was added. After a few minutes of stirring, a solution of *m*CPBA (34 mg, 0.15 mmol, 1.05 equiv.) in CH_2_Cl_2_ (10 mL) was added in several portions. The reaction was stirred at -10 ºC for 1 hour and quenched with water (5 mL) and Na_2_CO_3_ (5 mL, 1.0 M). The organic phase was collected, dried over Na_2_SO_4_ and the solvent removed under rotatory vacuum. The crude was purified by flash chromatography (eluent: CH_2_Cl_2_) to give **PDIb-SO** as a red solid (54 mg, 0.07 mmol, 50% yield). ^1^H NMR (500 MHz, CD_2_Cl_2_, 25 °C): δ (ppm) 9.08 – 9.01 (br, 2H, H_1_), 8.77 – 8.69 (br, 2H, H_2_), 8.63 (d, *J* = 7.9 Hz, 2H, H_3_), 5.23 – 5.14 (m, 2H, H_4_), 2.27 – 2.19 (m, 4H, H_5_), 1.90 – 1.81 (m, 4H, H_5_’), 1.35 – 1.21 (m, 24H, H_6_), 0.84 (t, *J* = 7.0 Hz, 12H, H_7_). Signals corresponding to H1 and H2 appear broad at room temperature due to the existence of slow conformational changes in the rigid aromatic scaffold. ^13^C NMR (125 MHz, CD_2_Cl_2_, 25 °C): δ (ppm) 145.4 (C_2_), 135.5 (C_5_), 134.9 (C_3_), 134.3 and 133.6 (C_7_), 130.8 (C_9_), 128.9 and 128.1 (C_1_), 125.6 (C_6_), 124.4 (C_4_), 55.4 (C_13_), 32.7 (C_14_), 32.1 (C_15_), 27.0 (C_16_), 23.0 (C_17_), 14.2 (C_18_). Signals corresponding to C_8_, C_10_, C_11_, and C_12_ are not detected due to conformational equilibrium, as well as proximity to the quadrupole nitrogen, as usually observed in PDIs. The two signals attributed to C_1_ and C_7_, respectively, stem from the slow conformational changes. HRMS (TOF-MS CI+): *m/z* calcd for C_46_H_52_N_2_O_5_S 744.3597, found: 744.3595 [M^+^]. The structure was also confirmed by X-ray diffraction (see section 7.3)

**H_7_**

**H_6_**

**H_5_**

**H_5_’**

**H_4_**

**H_1_**

**H_3_**

**H_2_**

**H_2_**

*Figure S3. ^1^H-NMR spectrum of* ***PDIb-SO****, in CD_2_Cl_2_ (500 MHz, 25 °C). The resolution of H_1_ and H_2_ peaks is affected by the existence of slow conformational changes in the rigid aromatic scaffold. In place of the expected singlet (H_1_) and doublet (H_2_), two broad peaks are observed at room temperature. In order to resolve these signals, variable temperature (VT)-^1^H-NMR was performed (see Figure S4).*

**25 ºC**

**35 ºC**

**45 ºC**

**55 ºC**

**65 ºC**

**75 ºC**

*Figure S4. VT-^1^H-NMR spectra (300 MHz) of* ***PDIb-SO*** *in tetrachloroethane-d2. The NMR sample was progressively heated and measured at temperatures ranging from 25 ºC to 75 ºC, and then cooled down again to room temperature to ensure the integrity of the compound. Above room temperature the broad peaks corresponding to H_1_ and H_2_ (see Figure S3) and located at 9.05 ppm and 8.73 ppm, respectively, start to evolve into a singlet and a doublet, as can be observed at 75 ºC. This can be ascribed to a faster interconversion of the different conformers.*

**C_7_**

**C_1_**

**C_6_**

**C_4_**

**C_2_**

**C_5_**

**C_3_**

**C_9_**

**C_13_**

**C_14_**

**C_15_**

**C_16_**

**C_17_**

**C_18_**

*Figure S5. ^13^C-Jmod-NMR spectrum of* ***PDIb-SO****, in CD_2_Cl_2_ (125 MHz, 25°C). Carbon peaks corresponding to C_8_, C_10_, C_11_ and C_12_, could not be detected due to the above-mentioned conformational equilibrium, as well as the proximity to the quadrupole nitrogen. This phenomenon observed in PDIs or other rigid blocks, such as porphyrinoids, is well known by the community. Additionally, the sets of two carbons peaks at 134.3 and 133.6 ppm, as well as 128.9 and 128.1 ppm, can be attributed to C_7_ and C_1_, respectively, stemming from the slow conformational changes.*

**H_1_-C_1_**

**H_3_-C_6_**

**H_2_-C_7_**

**H_4_-C_13_**

**H_5_/H_5_’-C_14_**

**H_6_-C_15_/C_16_/C_17_**

**H_7_-C_18_**

*Figure S6. HSQC spectrum of* ***PDIb-SO****, in CD_2_Cl_2_ (25 °C).*

*Figure S7. High Resolution Mass Spectrometry spectrum of* ***PDIb-SO*** *(TOF-MS CI+).*

# 3. Optical characterization

*Figure S8. Normalized UV-vis absorption spectra of* ***PDIa-S****,* ***PDIa-SO****,* ***PDIb-S*** *and* ***PDIb-SO*** *in CH_2_Cl_2_ (10^-5^ M).*

*Figure S9. Normalized UV-vis emission spectra of* ***PDIa-S****,* ***PDIa-SO****,* ***PDIb-S*** *and* ***PDIb-SO*** *in CH_2_Cl_2_ (10^-5^ M).  λ_ex_ = 495 nm for PDI-S derivatives and 535 nm for PDI-SO compounds.*

*Figure S10. a) Deoxygenation of a* ***PDIb-SO*** *thin-film on glass upon visible light irradiation during 165 min, monitored by UV-vis absorption. Similarly to the experiment in CH_2_Cl_2_ solution, the photoinduced deoxygenation can be monitored by the modification on the structured bands of the perylene. Noteworthy, the band at ca. 550 nm disappears, while the transitions located at higher energies (400-500 nm) increase in intensity. b)* ***PDIb-S*** *thin-film absorption spectra before and after 8 hours under visible light irradiation. The spectra show minimal changes confirming the photostability of the material. c) Kinetic profiles of the photochemical deoxygenation of* ***PDIb-SO*** *spin-coated onto glass substrates from chloroform solutions. Different thicknesses were achieved (Figure S29) using 10, 20 and 30 mg/mL solutions. Regardless of the thin film thickness, the deoxygenation reaction to give* ***PDIb-S*** *showed similar pseudo first order profiles and reaction rates, reaching completion after ca. 3 hours under a photoreactor irradiation. d)* ***PDIb-SO*** *thin-film absorption spectra stability tests under ambient light conditions. Spectral modifications after 1 week and 1 month suggest that slow deoxygenation takes place under ambient light and, although the patterns on the films are visible, encapsulation of the signatures might be considered.*

# 4. Theoretical calculations

The density functional theory (DFT) geometry optimization was carried out with Gaussian16^[4]^ at the PBE1PBE/6-311+g(d,p) level applying the D3 version of Grimme’s dispersion and applying the polarizable continuum mode (PCM) using a screening dielectric constant, with the role of solvent polarity being addressed elsewhere. The absorption spectra were computed with full TD-DFT calculations at the ωB97X-D/6-31G(d,p)/D3/PCM. Molecular orbitals were illustrated with the software IQmol.

Computed NMR shifts were determined using the GIAO method with Gaussian16^[4]^ at the WP04/cc-pVDZ level of theory and they were empirically scaled following the reported method of Tantillo *et al.*^[5,6]^ The ^1^H-^1^H coupling constants were simulated at the B3LYP/6-31G(d,p) level of theory. In both cases, the influence of chloroform was included by the PCM model. ^1^H-NMR shifts and coupling constants were extracted using scripts available at the CHEmical SHIft REpository (CHESHIRE).^[7]^ The simulated ^1^H-NMR spectra were generated in MestReNova 14.0.1 using the spin simulation tool. ^13^C-NMR shifts and spectra were extracted using the software Multiwfn^[8,9]^ and eventually depicted with Origin.

For simplicity and computing time economy, calculations were performed on a model system (**PDI-SO**) displaying methyl groups as *N*-substituents, instead of the cyclohexyl and 6-undecyl moieties present in **PDIa-SO** and **PDIb-SO**, respectively.

a)

| b) | **PDI-S** | **PDI-SO** |
| --- | --- | --- |
| LUMO |   - 3.56 ev  π = 82% |   -3.89 ev  π = 78% |
| HOMO |   -6.50 ev  π = 100% |   -6.69 ev  π = 99% |

*Figure S11. a) TD-DFT simulated UV-vis spectra of model systems* ***PDI-S*** *(black curve) and* ***PDI-SO*** *(red curve). Vertical lines correspond to the excited state transitions and the representative oscillator strength. b) HOMO-LUMO distributions of* ***PDI-S*** *and* ***PDI-SO*** *and π character of each orbital. Theoretical calculations confirm the π-π* properties of the first excited state transition.*

*Table S2. Contribution of the optical transitions obtained from TD-DFT calculations for model compounds* ***PDI-S*** *and* ***PDI-SO****.*

| Compound | λ_maxABS_ | Oscillator Strength | Major contribution | Minor contribution |
| --- | --- | --- | --- | --- |
| PDI-S    PDI-SO | @490  @416 @329  @315  @530  @476  @363  @323 | 0.84  0.14  0.01  0.05  0.70  0.01  0.03  0.12 | HOMO → LUMO  HOMO-1 → LUMO  HOMO → LUMO+1  HOMO-6 → LUMO  HOMO → LUMO  HOMO-1 → LUMO  HOMO-4 → LUMO  HOMO-9 → LUMO | -  H → L+4  H-6 → L; H-7 → L  H-7 → L; H → L+1  -  -  H-6 → L; H-2 → L  H-11 → L; H-7 → L |

**H_1_**

**H_3_**

**H_2_**

*Figure S12. Aromatic region of the calculated ^1^H-NMR (0.5 Hz line width) spectrum of* ***PDI-SO****.*

*Figure S13. 120-180 ppm region of the calculated ^13^C-NMR spectrum of* ***PDI-SO****.*

# 5. Anti-counterfeiting photopatterns

## 5.1 Handwritten photomask

Handwritten photomasks were prepared using a clean glass substrate of dimensions 2 x 1.5 cm^2^ and a standard dark (blue or black) permanent marker (ultra fine point - 0.8 mm). Goya’s signature (Figure S14, right) was carefully reproduced on top of the substrate making use of the mentioned pen. Once the ink was totally dry, the mask could be used on top of the **PDIb-SO** thin-films to stamp the signature upon photoirradiation (400-600 nm) without compromising the integrity of the mask.

*Figure S14. Handwritten photomask prepared in our lab (top left) and the used signature of reference from the painter Francisco de Goya (top right). Schematic representation of the photopatterning process using handwritten a mask.*

## 5.2 Stereolithography 3D printing (polymer-based photomask)

Designs were drawn on Fusion 360 (Autodesk) and converted into .stl files. These files were then sliced using proprietary softwares Nauta and Fictor (DWS Systems) and printed using a DWS 029J+ printer (DWS Systems) with an opaque nano-filled ceramic resin (Precisa DL260, DWS Systems). This equipment has featured a minimum resolution of 200 µm, however, to obtain well resolved straight lines after irradiation, we were limited to 500 µm of line width. Completed prints were detached from the platform and developed using isopropanol or acetone.

*Figure S15. a) Computer design of the 3D mask containing the logo of the CEMES-CNRS laboratory. b) 3D printed mask (2.6 cm x2.0 cm) obtained by stereolithography from the previous design. c) Picture of the CEMES-CNRS logo engraved on a* ***PDIb-SO*** *thin-film on glass upon light irradiation (400-600 nm) using the 3D printed mask.*

## 5.3 Micrometric patterns

*Figure S16. Optical micrographs of different size patterns obtained through a chromium photomask in* ***PDIb-SO*** *thin-films deposited onto glass and irradiated at 400-600 nm.*

*Figure S17. Micrographs overlay of two* ***PDIb-SO*** *photopatterns observed by fluorescence microscopy.* ***PDIb-SO*** *was spin-coated onto glass a) from a 30 mg/mL chloroform solution, and b) from a 10 mg/mL chloroform solution.*

*Figure S18. Micrographs of* ***PDIb-SO*** *motifs obtained by photopatterning spin-coated thin-films on glass (30 mg/mL in chloroform) with the PRIMO equipment (left) and schematic representation of the process (right).*

## 5.4 Fabric patterning

A piece of cotton fabric extracted from a clean lab coat was dyed with **PDIb-SO** via the dip dyeing process (Figure S19). To do so, a solution of the synthetic red dye **PDIb-SO** (ca. 5 mg/mL) in EtOH/PhMe (9:1) was poured in a wide beaker and the white fabric sample immersed in the solution for 10 sec. The recovered red fabric was dried in air (under the fumehood) for 24 h. Thereafter, a selected region of cotton covered with the CEMES 3D printed mask (Figure 3) was irradiated with the HAMAMATZU UV Spot Light Source (400-600 nm) overnight.

*Figure S19. Illustrated dip dyeing process of the cotton fabric.*

## 5.5 OFET prototype patterning

To test the potential application of the **PDIb-SO** patterning in electronic devices we first fabricated a bottom gate – bottom contact OFET prototype using the perylene derivative as organic semiconductor. To prepare the latter device, Au (50 nm) was deposited under reduced pressure using a shadow mask onto highly n-doped silicon wafers with a 300 nm-thick thermally grown SiO_2_ layer. Finally, **PDIb-SO** was spin-coated (6000 rpm) on top from a chloroform solution (30 mg/mL).

Using a chromium mask, on top of the device, we addressed the photoprinting process on the **PDIb-SO** thin-film.

*Figure S20. a) Schematic representation of the OFET structure with* ***PDIb-SO*** *as organic semiconductor. b) Picture of the fabricated prototype.*

# 6. Swab test

To enhance the security level of the anti-counterfeiting signatures, the material patterns can be analyzed by mass spectrometry. Being composed solely of the pure molecular precursor and of its photodeoxygenated derivative, the expected response should match the most abundant isotopes of **PDIb-SO** and **PDIb-S**. As depicted in Figure S21, a cotton swab was rubbed on the material containing the printed pattern. The swab head was cut off and recovered in an Eppendorf tube, where the cotton sample was extracted with CH_2_Cl_2_.The resulting organic solution was analyzed by MALDI-TOF.

*Figure S21. Schematic illustration of the swab test verification.*

[M+2O]^-^

[M+O]^-^

[M-O]^-^

[M]^-^

*Figure S22. Low-Resolution Mass Spectrometry (MALDI-TOF-) spectrum obtained from a swab test of a non-irradiated* ***PDIb-SO*** *film. The peak at 744.4 m/z corresponds to the [M]^-^ ion* ***PDIb-SO****, while 728.4, 760.4 and 776.4 m/z signals can be assigned to [M-O]^-^, [M+O]^-^ and [M+2O]^-^ species, formed upon ionization.*

[M+3O]^-^

[M+2O]^-^

[M+O]^-^

[M]^-^

*Figure S23. Low-Resolution Mass Spectrometry (MALDI-TOF-) spectrum obtained from a swab test of a photopatterned (400-600 nm)* ***PDIb-SO*** *film. The peak at 728.4 m/z corresponds to the [M]^-^ ion* ***PDIb-S****, while 744.4, 760.4 and 776.4 m/z match the mass of [M+O]^-^, [M+2O]^-^ and [M+3O]^-^, adducts obtained upon ionization.*

# 7. Solid state characterization

## 7.1 Optical and polarized optical microscopy

*Figure S24. Optical (right image) and polarized optical (left image) microscopy of spin-coated* ***PDIb-SO*** *thin-films from chloroform (30 mg/mL) on glass before (a) and after (b) irradiation (400-600 nm, 30 min)*.

## 7.2 Thermogravimetric analysis

*Figure S25. Thermogravimetry analysis (TGA) of compound* ***PDIb-SO****. The thermogram shows that the sample is stable until 290 ºC, thereafter it degrades until ca. 440 ºC with a weight loss of 38% and after a plateau, continues with another 20% loss until 600 ºC.*

## 7.3 Single crystal X-ray diffraction

Crystals suitable for XRD analysis were obtained by slow diffusion of hexane in a **PDIb-SO** chloroform solution. A red, block-shaped crystal was then mounted on a MiTeGen micromount with perfluoroether oil. Data for **PDIb-SO** were collected from a single crystal at 193(2) K on a Bruker D8 QUEST KAPPA diffractometer with a microfocus sealed tube using a multilayer mirror as monochromator and a Bruker PHOTON III CPAD detector. The diffractometer was equipped with an Oxford Cryostream 700 low temperature device and used MoKα radiation (λ = 0.71073 Å). All data were integrated with SAINT V8.41, yielding 106431 reflections of which 14247 were independent and 69.1% were greater than 2σ(*F^2^*).^[10]^ A Multi-Scan absorption correction using SADABS 2016/2 was applied.^[11]^ The structure was solved by Intrinsic Phasing methods with XT, VERSION 2018/2 and refined by full-matrix least-squares methods against *F^2^* using SHELXL-2019/2.^[12,13]^ All non-hydrogen atoms were refined with anisotropic displacement parameters. All hydrogen atoms were refined isotropic on calculated positions using a riding model with their Uiso values constrained to 1.5 times the Ueq of their pivot atoms for terminal sp3 carbon atoms and 1.2 times for all other carbon atoms. Crystallographic data for the structure reported in this paper have been deposited with the Cambridge Crystallographic Data Centre.^[14]^

Some moieties of the structure were found to be disordered. Several restraints (SAME, SIMU, DELU, RIGU, ISOR) and equal xyz and U_ij_ constraints (EXYZ and EADP) were applied to refine these disordered moieties and to avoid the collapse of the structures during the least-squares refinement by the large anisotropic displacement parameters.

CCDC 2517519 contains the supplementary crystallographic data for this paper. These data can be obtained free of charge from The Cambridge Crystallographic Data Centre via www.ccdc.cam.ac.uk/structures. This report and the CIF file were generated using FinalCif (<https://dkratzert.de/finalcif.html>).

*Figure S26. Picture of the crystals of compound* ***PDIb-SO****.*

a)

b)

*Figure S27. Molecular top view (a) and side view (b) of the molecular structure of* ***PDIb-SO****. Thermal ellipsoids are drawn at 30% probability level. Hydrogen and disordered atoms are omitted for clarity.*

*Table S3. Crystal data and structure refinement of* ***PDIb-SO****.*

| CCDC number | 2517519 |
| --- | --- |
| Empirical formula | C_46_H_52_N_2_O_5_S |
| Formula weight | 744.95 |
| Crystal system | triclinic |
| Space group (number) | $P\overline{1}$ (2) |
| *a* [Å] | 8.1507(10) |
| *b* [Å] | 18.581(2) |
| *c* [Å] | 25.449(3) |
| α [°] | 91.580(4) |
| β [°] | 98.859(5) |
| γ [°] | 90.274(5) |
| Volume [Å^3^] | 3806.7(8) |
| *Z* | 4 |
| *ρ*_calc_ [gcm^−3^] | 1.3 |
| *μ* [mm^−1^] | 0.136 |
| *F*(000) | 1592 |
| Crystal size [mm^3^] | 0.18×0.2×0.2 |
| Crystal colour | red |
| Crystal shape | block |
| Reflections collected | 106431 |
| Independent reflections | 14247  *R*_int_ = 0.0676 *R*_sigma_ = 0.0477 |
| Completeness to θ = 25.242° | 99.9 |
| Data / Restraints / Parameters | 14247 / 1490 / 1285 |
| Goodness-of-fit on *F*^2^ | 1.074 |
| Final *R* indexes  [*I*≥2σ(*I*)] | *R*_1_ = 0.0940 w*R*_2_ = 0.2336 |
| Final *R* indexes  [all data] | *R*_1_ = 0.1336 w*R*_2_ = 0.2699 |
| Largest peak/hole [eÅ^−3^] | 0.40/−0.54 |

## 7.4 X-ray photoelectronic spectroscopy (XPS)

XPS measurements were carried out in a Versaprobe III AD Physical Electronics (ULVAC) equipment using a monochromatic source Al Kα (1486.7 eV). A preliminary analysis was performed to determine the present elements (wide scan: step energy 0.2 eV, pass energy 224 eV) and consequently the detected elements were analysed in detail (detail scan: step energy 0.05 eV, pass energy 27 eV, time per step 20 ms) with an exit angle of 45º. The spectrometer was previously calibrated with Ag (Ag 3d5/2, 368.26 eV). The spectra were plotted using CasaXPS 2.3.26 software modelling the contributions after a background subtraction (Shirley). C1s was use as charge reference.

*Figure S28. XPS spectra of a* ***PDIb-SO*** *thin-film spin coated onto glass from a chloroform solution (30 mg/mL). a) Survey spectrum of the material in the initial state (before irradiation) and b) S2p spectra of the thin film before (bottom) and after (top) irradiation.*

The survey spectrum of the PDIb-SO thin-film showed peaks at S2p, C1s, N1s and O1s regions (Figure S28.a). Detailed analysis of the S2p region (160-175 eV) displayed the characteristic spin-orbit doublet (S2p_3/2_ and S2p_1/2_) with a 2:1 ratio and splinting of ca. 1.17 eV (Figure S28.b, bottom). First bands at 164.2 and 165.4 eV are ascribed to a thioether (Ar-S-Ar) chemical state, while prominent peaks at 166.1 and 167.3 eV (depicted in grey) can be attributed to the sulfoxide moiety and the less intense bands at 168.1 and 169.3 eV are characteristic of a higher oxidation state at sulfur. The presence of signals at lower and higher binding energies with respect to the sulfoxide moiety is ascribed to beam damage of the material during XPS experiments, resulting especially in partial deoxygenation, as previously observed with dibenzothiophene sulfoxide analogues.^[15]^ Importantly, the integrity of the thin films was assessed by UV–Vis spectroscopy prior to XPS analysis, which confirmed the absence of S-containing byproducts in the sample.

Comparison of the XPS spectra before and after irradiation (Figure S28.b) showed the disappearance of the SO peaks (166.1 eV S2p_3/2_, 167.3 eV S2p_1/2_), confirming the total photoconversion of **PDIb-SO** in the thioether counterpart. Furthermore, new bands were not detected in the S2p region after irradiation, in agreement with the absence of new bonds in the thioether bridge.

## 7.5 Atomic force microscopy (AFM) analysis

AFM (Park System, NX10) was used to measure the thin film thicknesses by assessing the topological image of the thin-film scratch (Figure S29.a, b and c). **PDIb-SO** thin films spin-coated (6000 rpm, 60 sec) onto glass from three different solutions (10, 20 and 30 mg/mL in chloroform) were scratched with a stainless-steel tool without damaging the substrate, displaying an average thickness of 52, 78, and 172 nm, respectively (Figure S29.d). These results were subsequently used to evaluate the existence of photoconversion rate dependence and to proof the quantitative conversion of the material regardless of the thickness (Figure S10.c).

*Figure S29. a-c) AFM micrographs of* ***PDIb-SO*** *thin films spin-coated from chloroform solutions of a) 10 mg/mL, b) 20 mg/mL and c) 30 mg/mL and thickness profiles of the scratch performed on them. d) Graph representation of the average thickness vs. solution concentration employed for the fabrication of the* ***PDIb-SO*** *thin films.*

# 8. References

[1]	Y. Ma, Z. Shi, A. Zhang, J. Li, X. Wei, T. Jiang, Y. Li, X. Wang, *Dyes Pigm.* **2016**, *135*, 41.

[2]	W. Fan, N. Liang, D. Meng, J. Feng, Y. Li, J. Hou, Z. Wang, *Chem. Commun.* **2016**, *52*, 11500.

[3]	F. Rey‐Tarrío, C. Atienza, L. Sánchez, *Small* **2025**, *21*, 2504415.

[4]	Gaussian 16, M. J. Frisch, G. W. Trucks, H. B. Schlegel, G. E. Scuseria, M. A. Robb, J. R. Cheeseman, G. Scalmani, V. Barone, G. A. Petersson, H. Nakatsuji, X. Li, M. Caricato, A. V. Marenich, J. Bloino, B. G. Janesko, R. Gomperts, B. Mennucci, H. P. Hratchian, J. V. Ortiz, A. F. Izmaylov, J. L. Sonnenberg, D. Williams-Young, F. Ding, F. Lipparini, F. Egidi, J. Goings, B. Peng, A. Petrone, T. Henderson, D. Ranasinghe, V. G. Zakrzewski, J. Gao, N. Rega, G. Zheng, W. Liang, M. Hada, M. Ehara, K. Toyota, R. Fukuda, J. Hasegawa, M. Ishida, T. Nakajima, Y. Honda, O. Kitao, H. Nakai, T. Vreven, K. Throssell, J. A. Montgomery, Jr., J. E. Peralta, F. Ogliaro, M. J. Bearpark, J. J. Heyd, E. N. Brothers, K. N. Kudin, V. N. Staroverov, T. A. Keith, R. Kobayashi, J. Normand, K. Raghavachari, A. P. Rendell, J. C. Burant, S. S. Iyengar, J. Tomasi, M. Cossi, J. M. Millam, M. Klene, C. Adamo, R. Cammi, J. W. Ochterski, R. L. Martin, K. Morokuma, O. Farkas, J. B. Foresman, and D. J. Fox, Gaussian, Inc., Wallingford CT, **2016**.

[5]	M. W. Lodewyk, M. R. Siebert, D. J. Tantillo, *Chem. Rev.* **2012**, *112*, 1839.

[6]	M. W. Lodewyk, C. Soldi, P. B. Jones, M. M. Olmstead, J. Rita, J. T. Shaw, D. J. Tantillo, *J. Am. Chem. Soc.* **2012**, *134*, 18550.

[7]	T. Cheshire, P. Ramblenm, D. J. Tantillo, M. R. Siebert, M. W. Lodewyk, CHEmical SHift REpository with Coupling Constants Added Too, http://cheshirenmr.info

[8]	T. Lu, *J. Chem. Phys.* **2024**, *161*, 082503.

[9]	T. Lu, F. Chen, *J. Comput. Chem.* **2012**, *33*, 580.

[10]	B. A. I. SADABS, *Bruker*, Vol. Madison, Wisconsin, USA, **2008**.

[11]	L. Krause, R. Herbst-Irmer, G. M. Sheldrick, D. Stalke, *J. Appl. Crystallogr.* **2015**, *48*, 3.

[12]	G. M. Sheldrick, *Acta Crystallogr. C Struct. Chem.* **2015**, *71*, 3.

[13]	G. M. Sheldrick, *Acta Crystallogr. A Found. Adv.* **2015**, *71*, 3.

[14]	C. R. Groom, I. J. Bruno, M. P. Lightfoot, S. C. Ward, *Acta Crystallogr. B Struct. Sci. Cryst. Eng. Mater.* **2016**, *72*, 171.

[15]	M. Hankache, *et al.* Self-assembly and bimodal chemical activation of dibromo-dibenzothiophene-sulfoxide on Au(111), *submitted manuscript.*
